# Medical History from the Earliest Times

**Published:** 1893-02-11

**Authors:** E. T. Withington


### Medical History from the Earliest Times.

By E. T. Withington, M.A., M.B. Oxon.

### XLII.—THE MEDIÆVAL PHYSICIAN.

When King Gram went in disguise to a wedding for the purpose of carrying off the bride, he dressed in the dirtiest rags he could find, sat among the lowest menials in the hall, and called himself a physician. This is a sad contrast to the curled and scented dandies of the days of Aristophanes, but it is only one of many signs of the low social state of the profession during the early middle ages. Nor are the causes for this decline hard to discover. When the Christian Emperors, Valens and Valentinian, handed over the pagan Oribasius to "the most savage of the barbarians," in the pious hope that he would become a target for their arrows, they were disappointed to hear that his medical skill had raised him to the rank of a chief. But the physicians of the West could compare neither socially nor professionally with Oribasius; they still belonged largely to the servile class, and their skill, whether in medicine or surgery, was not sufficient to impress the rude conquerors, who looked upon the free-born Romans themselves as little better than slaves. Medicine was, indeed, saved from entire degradation by its connection with the Church, which did so much to soften the horrors of the barbarian conquest; but the suspicion with which its practitioners were regarded is very clearly shown by the laws passed concerning them, as in the following examples from the Visigothic code.

"No 'medicus' shall bleed a free woman except in the presence of a relative, or in case of necessity, of a respectable neighbour or servant, on pain of a fine of 10 solidi." "No medicus shall visit a prisoner unless accompanied by an official, for he may bring him a poison, and so defeat the ends of justice." "Before undertaking a case he shall enter into a contract and give pledges." "If a physician injures a freeman by bleeding, let him pay 100 solidi; but if the patient dies, let him be handed over to his relatives to treat as they please. If a slave is injured or killed, the physician shall replace him by one of equal value." This last law is especially important; it reappears in yet stronger terms in later codes, and was doubtless frequently put in force, and sometimes even exceeded. The plague which ravaged France in the sixth century attacked, among others, Austragild, wife of Guntram, King of the Burgundians, "a good and God-fearing prince." Before her death she begged her husband, and adjured him by the holy sacrament, to execute her two physicians, Nicholas and Donatus, on her tomb. The King did so, "but (adds Bishop Gregory) many considered that the deed was not without sin." In 1336, some time before John of Bohemia went to fight and fall at Crecy, a travelling oculist offered to cure him of his blindness. He failed, and was promptly thrown into the river Oder.

One result of the law was that practitioners refused to undertake dangerous cases at all, unless either the patient agreed to consider himself dead already, or his friends gave surety that they should not be held responsible for failure. About A.D. 1250, a nobleman of Bologna was injured in the side so severely that a portion of his lung protruded through the wound. For some time no one could be found to undertake the case, till at last an appeal was made to the two most famous operators of the age, Hugh, of Lucca, and Roland, of Parma. Those intrepid surgeons, having obtained permission of the Bishop, and taken an oath from thirty of the patient's friends that no harm should happen to them, boldly excised the protruded lung and applied a consolidative powder. The patient recovered, and went on a pilgrimage to Jerusalem.

The close relation of mediæval medicine to the Church has already been noticed, but we must remember that there was always a distinct class of lay practitioners, and that the priestly "doctor" was usually looked upon with disfavour by his ecclesiastical superiors. The sixth canon of the Council of Rheims, 1131, declares that "An evil and detestable custom has arisen that monks and regular clergy, despising the rules of the blessed Benedict and Augustine, practise law and medicine for worldly profit." Law is bad enough, and those rash men who occupy themselves therewith are to be severely chastised. But medicine is worse; nor is money-making its only objection—"*Cumque impudicus oculus impudici cordis sit nuntius, illa de quibus loqui erubuit honestas non debet religio pertrectare.* Wherefore, by Apostolic authority, we forbid the continuance of this practice, and let the bishops, abbots, and priors who connive at such an enormity be degraded and excommunicate." This canon was repeated by the second general Lateran Council, 1139, and again at Montpellier in 1162, when it was applied to all religiosi. The fourth Lateran Council, 1215, forbade all subdeacons, deacons, and priests to practise that part of surgery which has to do with burning and cutting; and, finally, Pope Honorius III. prohibited all persons in holy orders from practising medicine in any form. The number of these decrees indicates their want of effect. Distinguished clerical surgeons, as, for instance, Theodoric and Guy of Chauliac, continued burning and cutting, in entire disregard of the oft-quoted "*ecclesia abhorret a sanguine*," and the final separation of medicine from the Church was probably due to more general causes, such as the increasing complexity of the art, the rise of the great secular universities, Salerno, Montpellier, Padua, and Bologna, and especially to the fact that the recognised medical textbooks were translations from the works of misbelieving Moslem, whose chief Christian exponents had been convicted of numerous and deadly heresies.

The most lively picture of the mediæval physician is given in a twelfth century treatise entitled "De Adventu Medici," or "The Doctor's Visit," and attributed to Archimatthaeus, one of the Salernitan masters. The following is a condensed abstract of this work, which, in spite of its quaint piety, contains so much of the wisdom of the serpent that it compares somewhat unfavourably with analagous passages by pagan physicians. "When called to a patient commend yourself to God and to the angel who guided Tobias. On the way learn as much as possible from the messenger, so that if you discover nothing from the patient's pulse or water, you may still astonish him and gain his confidence by your knowledge of the case. On arrival ask the friends whether the patient has confessed, for if you bid him do so after the examination it will frighten him. Then sit down, take a drink, and praise the beauty of the country and the house, if they deserve it, or extol the liberality of the family. Next proceed to feel his pulse, remembering that it may be affected by your arrival, or, the patient being a miser, by his thinking of the fee. Do not be in a hurry to give an opinion, for the friends will be more grateful for your judgment if they have to wait for it. Tell the patient you will cure him, with God's help, but inform his friends that the case is a most serious one. Look not desirously on the man's wife, daughter, or handmaid, for this blinds the eyes of the physician, deprives him of the divine assistance, and disturbs the patient's mind. If, according to custom, you are asked to dinner, do not hasten to take the first place, unless, as is usual for the priest and the physician, it is offered to you. Often send to inquire how the patient is, that he may see you do not neglect him for the pleasures of the table, and on leaving, express your thanks for the attention shown you, for this will please him much." Then come directions for treatment in simple cases, and finally the important question of the fee. "When the patient is nearly well, address the head of the family, or the sick man's nearest relative, thus: 'God Almighty having deigned by our aid to restore him whom you asked us to visit, we pray that He will maintain this health, and that you will now give us an honourable dismissal. Should any other member of your family desire our aid, we should, in grateful remembrance of our former dealings with you, leave all else and hurry to serve him." Another Salernitan suggests a terrible vengeance which may be inflicted on a nonpaying patient. "Contrive that he shall take alum instead of salt with his meat; this will not fail to make him come out all over spots."

